# Traditional serrated adenoma has two distinct genetic pathways for molecular tumorigenesis with potential neoplastic progression

**DOI:** 10.1007/s00535-020-01697-5

**Published:** 2020-06-13

**Authors:** Yoshihito Tanaka, Makoto Eizuka, Noriyuki Uesugi, Keisuke Kawasaki, Hiroo Yamano, Hiromu Suzuki, Takayuki Matsumoto, Tamotsu Sugai

**Affiliations:** 1 Department of Molecular Diagnostic Pathology, School of Medicine, Iwate Medical University, 2-1-1, Idai-dori, Yahaba-cho, Shiwa-gun, Iwate, 028-3695 Japan; 2 Division of Gastroenterology, Department of Internal Medicine, School of Medicine, Iwate Medical University, 2-1-1, Idai-dori, Yahaba-cho, Shiwa-gun, Iwate, 028-3695 Japan; 3 Department of Gastroenterology and Hepatology, School of Medicine, Sapporo Medical University, South-1 West-17, Chuo-ku, Sapporo, 060-8556 Japan; 4 Department of Molecular Biology, School of Medicine, Sapporo Medical University, South-1 West-17, Chuo-ku, Sapporo, 060-8556 Japan

**Keywords:** Annexin A10, *BRAF* mutation, *KRAS* mutation, DNA methylation, Traditional serrated adenoma

## Abstract

**Background:**

Recent studies have shown that traditional serrated adenoma (TSA) can be classified into BRAF and KRAS subtypes. Here, we examined the clinicopathological and molecular findings of 73 TSAs.

**Materials and methods:**

TSAs were subclassified into BRAF type (46 cases, type A) and KRAS type (27 cases, type B) and divided into polyp head (TSA component) and base (precursor component [PC]) to identify pathological and molecular differences between the two components. *BRAF* and *KRAS* mutations, microsatellite instability (MSI), and DNA methylation status of the TSA component and PC were analyzed. In addition, immunohistochemical expressions of annexin A10, MUC2, MUC5AC, MUC6, and CD10 were also examined. Finally, we compared endoscopic findings with histological features.

**Results:**

We classified type As into 31 type A1s with mutation of the corresponding PC (42.5%) and 15 type A2s without mutation of the PC (20.5%). None of the corresponding PCs without *KRAS* mutation were observed in type Bs. MSI was not detected in the TSAs examined. There were significant differences in the frequency of annexin A10 and MUC5AC expression between the three subtypes. Furthermore, we compared the TSA component with the corresponding PC to identify the progression mechanism between the two components. Methylation status played an important role in the progression of type A1 from the corresponding PC, unlike type A2 and type B. Finally, specific endoscopic findings were well correlated with distinct histological findings.

**Conclusion:**

TSAs were heterogeneous tumors with two or three pathways to neoplastic progression.

**Electronic supplementary material:**

The online version of this article (10.1007/s00535-020-01697-5) contains supplementary material, which is available to authorized users.

## Introduction

Although traditional serrated adenoma (TSA) is a rare type of serrated polyp that accounts for less than 1% of all colorectal polyps [1], TSA is now thought to be an important type of colorectal polyp occasionally encountered in routine histopathological diagnosis [2, 3]. TSA was initially described by Longacre et al. in 1990 [4]. However, the terminology was confusing for some time because the histological criteria for TSA were not standardized until studies by Torlakovic et al. [5, 6]. TSA is characterized by its predominance in the left-sided colon; a villiform-papillary and protruded growth pattern; serrated contours; and columnar lining cells with eosinophilic cytoplasm, pencil-like nuclei, and ectopic crypt foci, enabling TSA to be differentiated from other types of serrated polyps, such as sessile serrated lesions (SSLs), which were often mistakenly diagnosed as TSA in previous literature [7, 8].

According to traditional histological classification, serrated polyps are primarily classified into three subtypes, including hyperplastic polyps (HPs), TSA, and SSLs [7]. SSL is recognized as a type of precursor lesion for colorectal cancer (CRC) with a microsatellite instability (MSI)-high phenotype, whereas TSA is likely to progress into CRC with a microsatellite stable (MSS) phenotype [7–10]. However, TSA is a heterogeneous disease characterized by a phenotype exhibiting the molecular characteristics of both the serrated pathway and the adenoma–carcinoma sequence [7, 9–11]. Despite our knowledge of these molecular features, genetic alterations occurring during the tumorigenesis of TSA have not been fully evaluated.

Previous studies have shown that there are two subtypes of TSAs, i.e., BRAF and KRAS types [11–14]. Mutations in *BRAF* and *KRAS* lead to common signaling pathway alterations, resulting in activation of the EGFR signaling pathway [15]. However, the detailed molecular differences in TSA tumorigenesis based on the presence/absence of *BRAF* and *KRAS* mutations remain unknown. In the current study, we examined a series of TSAs with *BRAF* or *KRAS* mutations, focusing on the molecular genetics associated with the serrated pathway, including annexin A10, mucin marker, and CD10 expression; DNA methylation status; and clinicopathological features. The aim of this study was to identify heterogeneity in the molecular events of the neoplastic pathway according to the mutation status. In addition, we evaluated molecular alterations occurring during the progression of TSA.

## Materials and methods

### Patients

**Fig. 1:**
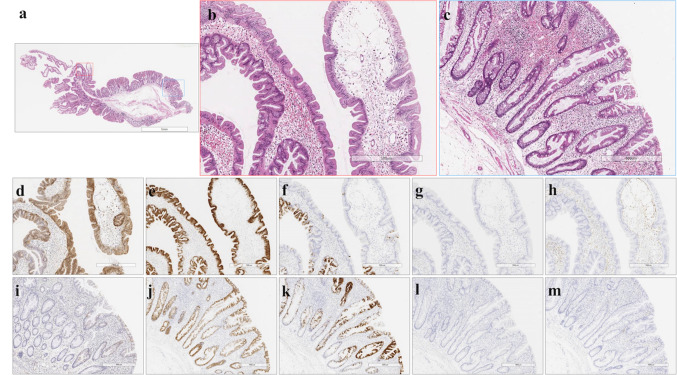
Histological and immunohistochemical staining of type A1 TSAs. **a** HE staining. **b** A high-power view showing the TSA component (red box in Fig. 1a). **c** A high-power view showing the precursor component (blue box in Fig. 1a). **d** Positive for annexin A10 expression. **e** Positive for MUC2 expression. **f** Partially positive for MUC5AC expression. **g** Negative for MUC6 expression. **h** Negative for CD10 expression. **i** Negative for annexin A10 expression. **j** Positive for MUC2 expression. **k** Positive for MUC5AC expression. **l** Negative for MUC6 expression. **m** Negative for CD10 expression

**Fig. 2:**
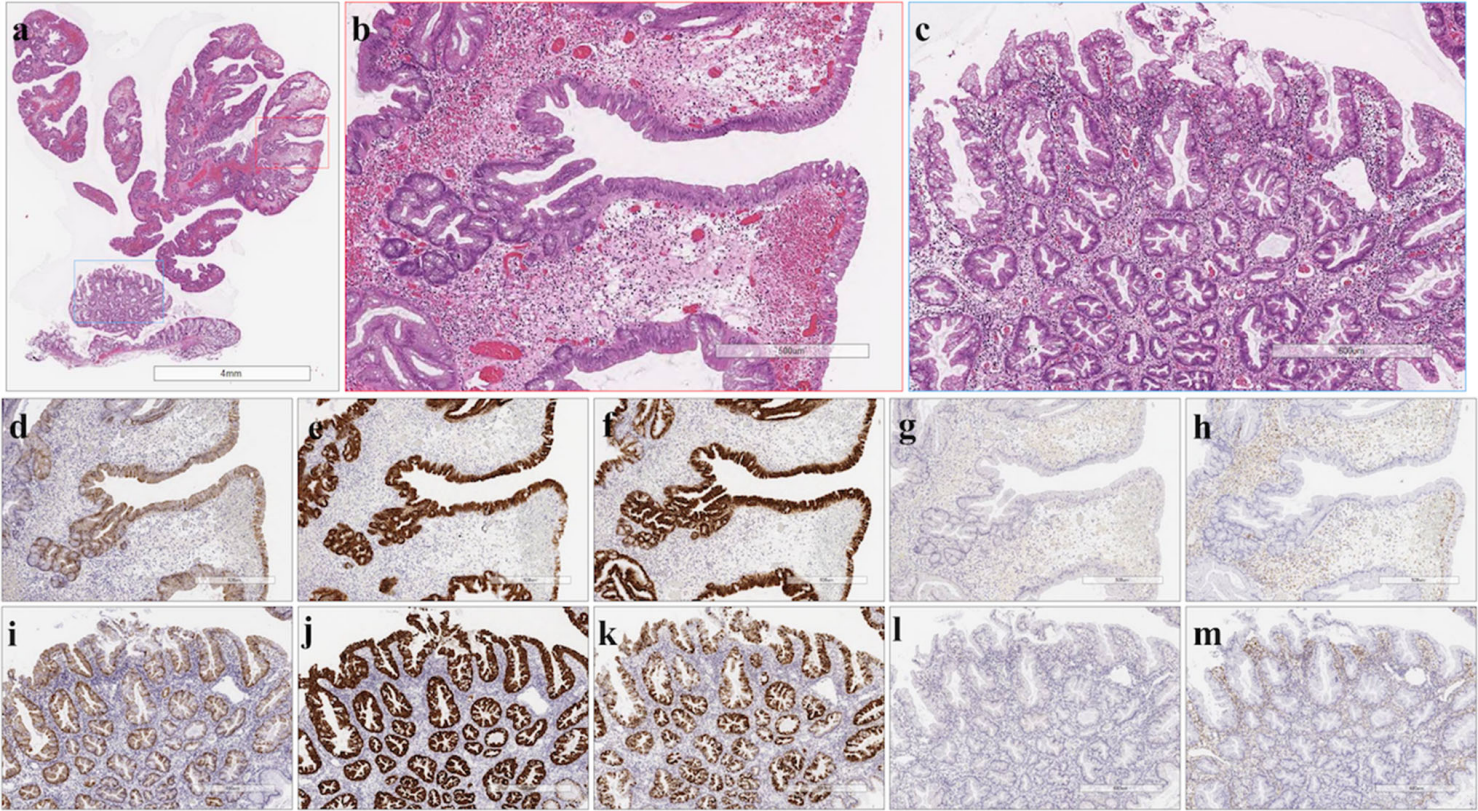
Histological and immunohistochemical staining of type A2 TSAs. **a** HE staining. **b** A high-power view showing the TSA component (red box in Fig. 2a). **c** A high-power view showing the precursor component (blue box in Fig. 2a). **d** Positive for annexin A10 expression. **e** Positive for MUC2 expression. **f** Positive for MUC5AC expression. **g** Negative for MUC6 expression. **h** Negative for CD10 expression. **i** Positive for annexin A10 expression. **j** Positive for MUC2 expression. **k** Positive for MUC5AC expression. **l** Negative for MUC6 expression. **m** Negative for CD10 expression

**Fig. 3:**
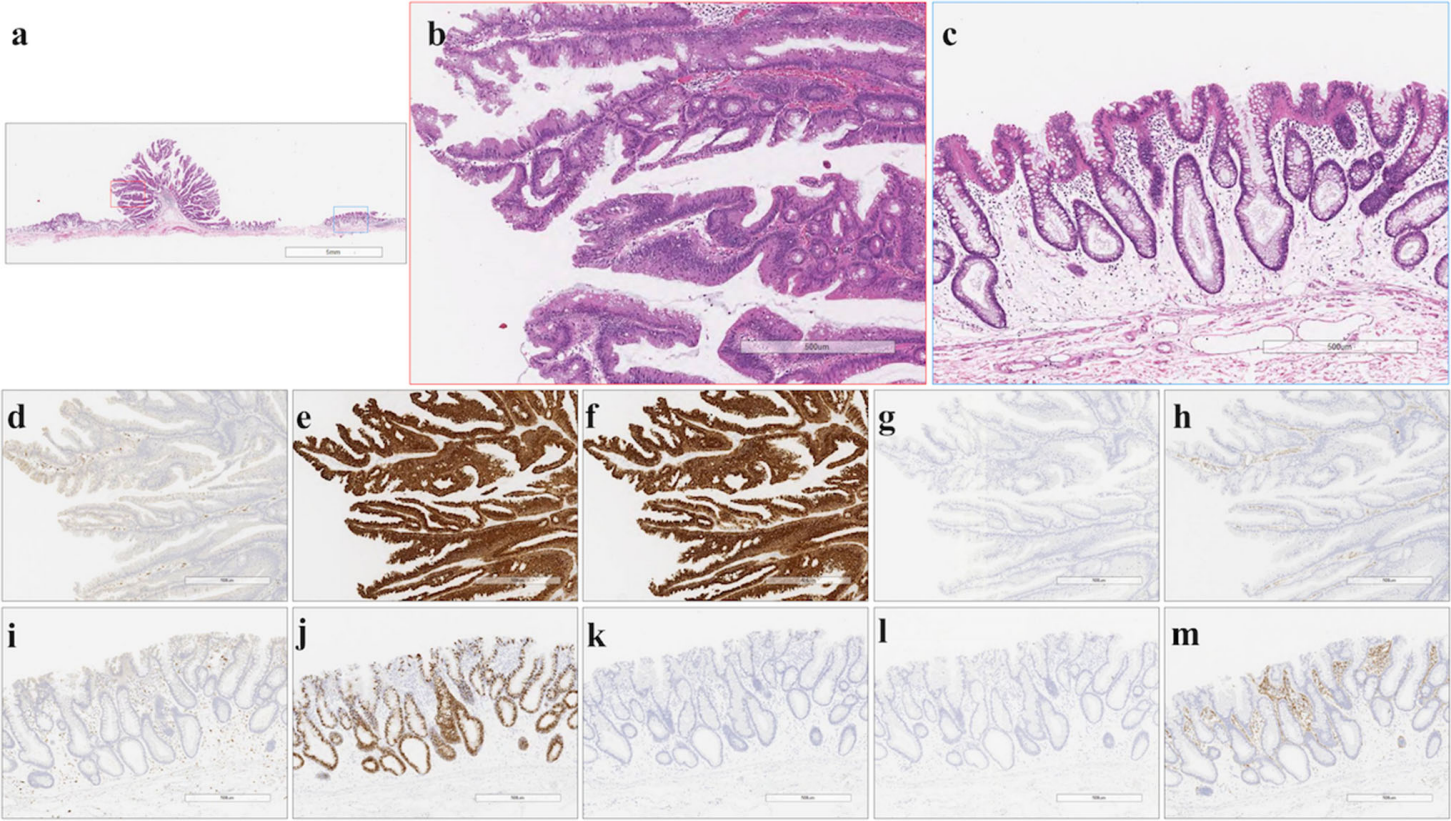
Histological and immunohistochemical staining of type B TSAs. **a** HE staining. **b** A high-power view showing the TSA component (red box in Fig. 3a). **c** A high-power view showing the precursor component (blue box in Fig. 3a). **d** Negative for annexin A10 expression. **e** Positive for MUC2 expression. **f** Positive for MUC5AC expression. **g** Negative for MUC6 expression. **h** Negative for CD10 expression. **i** Negative for annexin A10 expression. **j** Positive for MUC2 expression. **k** Negative for MUC5AC expression. **l** Negative for MUC6 expression. **m** Negative for CD10 expression

We reviewed 73 TSAs diagnosed from January 2014 to March 2018 that fulfilled the World Health Organization (WHO) criteria [7]. For reliable histological diagnosis, we used detailed criteria for TSA, including (1) papillary or villous surface mucosa; (2) dysplastic cells with pencil-like nuclei and eosinophilic cytoplasm; and (3) ectopic crypt foci (or crypt budding) and lesions associated with other histological components, such as SSLs [2, 7]. In the current study, adenomatous lesions (tubular, tubulovillous, and villous adenomas) were excluded. Two pathologists with expertise in gastrointestinal pathology independently reviewed these 73 TSAs. The histological feature was regarded as present if two pathologists agreed. In addition, TSA was divided into the two components, i.e., the head (TSA component) and base (precursor component) of the polyp. Representative results for TSAs are shown in Figs. 1, 2 and 3.

The study was approved by the research ethics committee of Iwate Medical University Hospital. Informed consent was obtained from all patients with TSA (HG 2018-012).

### Immunohistochemistry

Immediately after excision, the specimens were fixed in 10% neutral buffered formalin, embedded in paraffin wax, cut into 3-μm-thick paraffin sections, and stained with hematoxylin and eosin for routine pathological diagnosis. For immunohistochemical staining, additional 3-μm-thick sections were also cut from paraffin-embedded tissues and placed on poly-l-lysine-coated glass slides. To evaluate the expression of mucins and annexin A10, immunostaining was performed using antibodies against MUC2 (Ccp58; Novocastra Laboratories, Newcastle, UK), MUC6 (CLH5; Novocastra Laboratories), MUC5AC (CLH2; Novocastra Laboratories), annexin A10 (NBP1-90156; Novus, Litteleton, CO, USA), and CD10 (56C6; Novocastra Laboratories).

Immunohistochemistry was performed using the DAKO Envision + system, consisting of dextran polymers conjugated with horseradish peroxidase (DAKO, USA). The specimens were heated in citrate buffer (pH 6.0) using a microwave (H2500, Microwave Processor; Bio-Rad, USA) at 750 W three times for 5 min each before incubating with the antibodies, as described previously. Hematoxylin was used as the counterstain.

### Evaluation of mucin, CD10, and annexin A10 expression

Cytoplasmic expression of MUC2, MUC5AC, and MUC6 was regarded as positive immunostaining for these proteins, whereas CD10 immunostaining was considered positive if expression was observed along the brush border of tumor cells. Annexin A10 was regarded as positive if cytoplasmic and/or nuclear staining was detected. The proportion of positive tumor cell staining was graded as follows: 0 (negative), < 33% (1 +), 33–66% (2 +), and 66–100% (3 +). The staining intensity of tumor cells was graded as weak (1 +), moderate (2 +), or strong (3 +). Finally, the positive tumor cell score was determined by adding the proportion of the positive tumor cells and the staining intensity of tumor cells.

The presence of annexin A10 nuclear/cytoplasmic staining in more than 5% of the tumor area in any tissue section was defined as positive expression of annexin A10, in accordance with a previous report [16].

### DNA extraction

Microdissection of formalin-fixed, paraffin-embedded tissues was performed on hematoxylin-stained slides for three components, including head lesions, base lesions, and non-neoplastic mucosa. The three components were microdissected separately. Briefly, the tissue blocks were scratched at both the lateral margin of the tumor tissue to mark the surface of the target tissue. Subsequently, histological sections were removed from the histological block (up to 10 µm). We confirmed that the histological sections contained at least 50% tumor tissue. Microdissected tissue was incubated at 56 °C for 12–18 h in 50 µL buffer containing 0.5% Tween-20 (Boehringer Mannheim, Mannheim, Germany), 20 µg proteinase K (Boehringer Mannheim), 50 mM Trizma base (pH 8.9), and 2 mM ethylenediaminetetraacetic acid. Proteinase K was inactivated by incubating the samples at 100 °C for 10 min.

### Analysis of MSI

Polymerase chain reaction (PCR) analysis of MSI was performed as described previously [17]. Five different loci were assessed for MSI, including those recommended by the Bethesda panel for colon cancer (BAT25, BAT26, D5S346, D2S123, and D17S250). A tumor was defined as MSI-positive when PCR amplification of a specific marker resulted in an abnormal-sized DNA band in the tumor sample compared with the normal sample. MSI-positive CRC samples were used as controls in this study and were divided into two groups: those with high-level instability (i.e., MSI at ≥ 20% of loci) and those with low-level instability (i.e., MSI at < 20% of loci), as described previously. Low-level MSI was considered MSS in this study.

### DNA methylation analysis

Pyrosequencing using the PyroMark Q24 system was performed to assess the DNA methylation status of selected markers. Primer sequences were designed using PyroMark Assay Design 2.0 software (Qiagen, Hulsterweg, The Netherlands). The levels of DNA methylation at six specific promoters, originally described by Yagi et al. [18, 19], were quantified. Methylation at the promoters of three markers (*RUNX3*, *MINT31*, and *LOX*) was analyzed, and those with at least two of these markers methylated were defined as highly methylated epigenotype (HME) tumors. The remaining tumors were screened for methylation at the promoters of three other markers (*NEUROG1*, *ELMO1, and THBD*), and those with at least two of these markers methylated were defined as intermediate methylation epigenotype (IME) tumors. Tumors not classified as HME or IME were considered to have the low methylation epigenotype (LME).

### Analysis of KRAS and BRAF mutations

Mutations in the *KRAS* (codons 12, 13, and 61) and *BRAF* (V600E) genes were examined using a PyroMark Q24 pyrosequencer, as described previously [20]. Each reaction contained 1 × PCR buffer, 1.5 mM MgCl_2_, 0.2 mM each dNTP, 5 pmol forward primer, 5 pmol reverse primer (biotinylated), 0.8 U HotStarTaq DNA polymerase (Qiagen), 10 ng template DNA, and dH_2_O to a final volume of 25 μL. Cycling conditions were as follows: 95 °C for 15 min; 38 cycles of 95 °C for 20 s, 53 °C for 30 s, and 72 °C for 20 s; and a final extension at 72 °C for 5 min, with holding at 8 °C. Following amplification, 10 μL biotinylated PCR product was immobilized on streptavidin-coated sepharose beads (Streptavidin Sepharose High Performance; GE Healthcare Bio-Sciences Corp., Piscataway, NJ, USA) and washed in 70% EtOH. The purified biotinylated PCR products were loaded into the PyroMark Q24 (Qiagen) using PyroMark Gold reagents (Qiagen) containing 0.3 μM of the sequencing primer and annealing buffer.

### Colonoscopy evaluation

Total colonoscopies were performed using a magnifying endoscope (CF-H260AZI, CF-HQ290ZI; Olympus Medical Systems, Tokyo, Japan). When a lesion was detected by endoscopic examination, the surface mucus was washed away, and indigo carmine dye was spread over the lesion. The endoscopic findings of polyps, including tumor size, anatomic location, gross appearance, color, pit pattern, and the presence of a mucus cap, were evaluated by three endoscopists (Y.T., K.K., and H.Y.).

We retrospectively reviewed endoscopy images and re-evaluated the color, mucous cap, and mucosal pit pattern of each lesion according to Kudo’s classification [21], with a modification proposed by Ishigooka et al. [22]. In addition to the conventional type II pit pattern according to Kudo’s classification, the surface architecture of the lesions was also broadly categorized into type II-Open (type II-O), type II-Long (type II-L), or type IV-Serrated (type IV-S) according to Ishigooka’s classification. If several pit patterns were present in a single lesion, the pit pattern that occupied the greatest area was regarded as the basic architecture.

### Statistical analysis

For three-subgroup statistical analysis, data were analyzed using JMP 10.0 software package (SAS Institute, Inc., Cary, NC, USA) with Bonferroni corrections. Data obtained for clinicopathological features (sex, macroscopic type, and location), immunohistochemical patterns (annexin A10, MUC2, MUC5AC, MUC6, and CD10), and methylation status based on each subgroup (type A1, type A2, and type B) were analyzed using Fisher exact tests.

In addition, for three-subgroup statistical analysis of age and size, we used Kruskal–Wallis tests to compare categorical data. The level of significance was *P* < 0.05. If statistical differences between three subgroups were found, statistical analyses between two groups were further performed using Fisher exact tests. Differences in age and size distributions among patients in the two groups were evaluated using Mann–Whitney *U* tests. Differences with *p* values of less than 0.05 were considered significant. For statistical analysis of two components, differences between the two components were evaluated using McNemar’s test.

## Results

In the current study, polyp head and base lesions were termed TSA and precursor components, respectively. We examined mutations in the *BRAF* and *KRAS* genes in the polyp head (TSA components) and corresponding polyp base lesion (precursor component) separately. First, TSA components in 73 TSAs were tested for mutations in V600E *BRAF* and codons 12, 13 and 61 of *KRAS*. As a result, examined TSAs were classified into BRAF (type A; 46 cases) and KRAS (type B; 27 cases) types, respectively. Next, the precursor components were also examined for *KRAS (codons 12, 13 and 61)* and the *BRAF* (V600E) mutations. BRAF type was subclassified into type A1 with *BRAF* mutations in the precursor components (31 cases) and type A2 without *BRAF* mutations in the precursor components (15 cases); none of the corresponding precursor components without *KRAS* mutations were observed in type B.

Codons 12, 13, and 61 were examined in *KRAS* mutation analysis. G12D (GGT → GAT) and G12V (GGT → GTT) were found in 14 (51.9%) and 9 (33.3%) of 27 type B TSAs examined in this study, respectively. In addition, G13D (GGC → GAC) was observed in 4 (14.8%) of 27 type B TSAs we examined. Finally, there were no differences in the mutation statuses of *KRAS* and *BRAF* between TSA and precursor components.

There were no cases that overlapped between *KRAS*-mutated and *BRAF*-mutated cases in the examined TSAs. Additionally, no MSI was detected in the current study.

### Histological findings of the precursor components in type A and B TSAs

Specific histological findings were detected in the polyp base (precursor component) of the TSAs examined in this study. Precursor components of type A exhibited elongated crypts extending from the surface to the muscularis mucosa, tapering from a broad luminal opening to a narrowed base, with crypt branching to some extent, suggesting a microvesicular HP. Such histological features were found in all 46 type A cases. However, there were no differences in histological features between type A1 and type A2 precursors. These histological features were consistent with microvesicular type HPs. In contrast, the precursor component of type B TSAs consisted primarily of straight, adenomatous glands, but showed serration confined to the superficial layer. Such histological patterns were considered representative of superficially serrated adenoma, as described by Hashimoto et al. [23]. These lesions did not meet the histological criteria for SSLs proposed by the WHO.

### Differences in the frequency of clinicopathological findings among type A1, A2, and B TSAs

The proportion of men was significantly higher for type A1 TSAs (24/31, 77.4%) than for type A2 TSAs (7/15, 46.7%) and type B TSAs (12/27, 44.4%). The median age was significantly lower for type A1 TSAs than for type B TSAs. Moreover, there were significant differences in median sizes between type A1 and A2 TSAs (type A1 > type A2) and between type A2 and B TSAs (type B > type A2). Finally, statistical differences in the frequency of “flat and elevated lesions” between type A1 and B TSAs (type B > type A1) and between type A2 and type B TSAs (type B > type A2) were also found. The results of clinicopathological findings are summarized in Table 1.

**Table 1:** Comparison of clinicopathological findings among type A1, type A2, and type B TSAs

		Type A1 (%)	Type A2 (%)	Type B (%)	*P* value
Total		31 (100)	15 (100)	27 (100)	
Sex	Man	24 (77.4)^‡, ‡^	7 (46.7)^‡^	12 (44.4)^‡^	0.0216
	Woman	7 (22.6)	8 (53.3)	15 (55.6)	
Age, years	Median (range)	59.0 (30–77)^†^	64.0 (44–80)	69.0 (43–84)^†^	0.0034
Size, mm	Median (range)	15 (5–30)^‡^	9 (3–17)^‡, ‡^	14 (6–42)^‡^	0.0175
Location	Proximal	5 (16.1)	3 (20.0)	1 (3.7)	0.1990
	Distal	26 (83.9)	12 (80.0)	26 (96.3)	
Macroscopic type	Flat + elevated	4 (12.9) *	2 (13.3) ^†^	17 (63.0)*^, †^	< 0.0001
	Elevated	27 (87.1)	13 (86.7)	10 (37.0)	

*TSA* traditional serrated adenoma

^*^p < 0.001; ^†^*p* < 0.01; ^‡^*p* < 0.05

### Differences in the frequency of annexin A10, mucin marker, and CD10 expression and the methylation status of the TSA component among type A1, A2, and B TSAs

Significant differences in the frequency of annexin A10 expression were observed among the three subgroups (type A1, type A2, and type B); the differences between type A1 and type B TSAs and between type A2 and type B TSAs were also significant. No differences in the frequency of methylation status were observed for each subtype. In summary, there were no significant differences in the frequency of mucin marker, and CD10 expression and methylation status among the three subgroups, as shown in Table 2.

**Table 2:** Comparison of immunohistochemical features and methylation status among type A1, type A2, and type B TSAs

		Type A1 (%)	Type A2 (%)	Type B (%)	P value
(a) TSA component					
Total		31 (100)	15 (100)	27 (100)	
Annexin A10	Negative	13 (41.9)†	6 (40.0)‡	22 (81.5)†, ‡	0.0039
	Positive	18 (58.1)	9 (60.0)	5 (18.5)	
MUC2	Negative	0 (0)	0 (0)	0 (0)	1.0000
	Positive	31 (100)	15 (100)	27 (100)	
MUC5AC	Negative	13 (41.9)	7 (46.7)	12 (44.4)	1.0000
	Positive	18 (58.1)	8 (53.3)	15 (55.6)	
MUC6	Negative	31 (100)	15 (100)	27 (100)	1.0000
	Positive	0 (0)	0 (0)	0 (0)	
CD10	Negative	31 (100)	15 (100)	27 (100)	1.0000
	Positive	0 (0)	0 (0)	0 (0)	
Methylation status	LME	8 (25.8)	6 (40.0)	7 (25.9)	0.6288
	IME	19 (61.3)	8 (53.3)	19 (70.4)	
	HME	4 (12.9)	1 (6.7)	1 (3.7)	
(b) Precursor component					
Total		31 (100)	15 (100)	27 (100)	
Annexin A10	Negative	25 (80.6)	7 (46.7)*	27 (100)*	< 0.0001
	Positive	6 (19.4)	8 (53.3)	0 (0)	
MUC2	Negative	0 (0)	0 (0)	0 (0)	1.0000
	Positive	31 (100)	15 (100)	27 (100)	
MUC5AC	Negative	4 (12.9)*	2 (13.3)*	20 (74.1)*, *	< 0.0001
	Positive	27 (87.1)	13 (86.7)	7 (25.9)	
MUC6	Negative	31 (100)	15 (100)	27 (100)	1.0000
	Positive	0 (0)	0 (0)	0 (0)	
CD10	Negative	31 (100)	15 (100)	27 (100)	1.0000
	Positive	0 (0)	0 (0)	0 (0)	
Methylation status	LME	19 (61.3)	13 (86.7) †	12 (44.4)†	0.0109
	IME	12 (38.7)	1 (6.7) †	14 (51.9)†	
	HME	0 (0)	1 (6.7)	1 (3.7)	

*TSA* traditional serrated adenoma; *LME* low methylation epigenotype; *IME* intermediate methylation epigenotype; *HME* high methylation epigenotype

^*^p < 0.001; ^†^*p* < 0.01; ^‡^*p* < 0.05

### Differences in the frequency of annexin A10, mucin marker, and CD10 expression and methylation status in the corresponding precursor component among type A1, A2, and B TSAs

Although significant differences in the frequency of annexin A10 and MUC5AC expression and DNA methylation status were observed in the three-group statistical analysis, differences in the frequency of DNA methylation status in the two-subgroup comparison reached significance (type A2 versus type B). In addition, the frequency of annexin A10 and MUC5AC expression was significantly higher in type A2 TSAs than in type B TSAs, and the frequency of MUC5AC expression was significantly higher in type A1 TSAs than in type B TSAs. The results are shown in Table 2.

### Differences in the frequencies of annexin A10, mucin marker, and CD10 expression and methylation status in type A1 TSAs between precursor and TSA components

There was a significant difference in the frequency of annexin A10 expression between precursor and TSA components (TSA component > precursor component). Additionally, the frequency of MUC5AC expression was significantly higher in the precursor component than in the TSA component. Finally, there was a significant difference in the methylation LME status between the precursor component and TSA component (precursor component > TSA component). The results are depicted in Table 3.

**Table 3:** Pathological and molecular differences between the polyp base and head for each TSA type

		Polyp base	Polyp head
Type A1	Histology	MVHP	TSA
	Mutation	*BRAF* ( +)	*BRAF* ( +)
	Annexin A10*	(−) > ( +)	(−) < ( +)
	MUC5AC*	(−) < ( +)	(−) < ( +)
	DNA methylation^†^	low > intermediate	low, high < intermediate
Type A2	Histology	MVHP	TSA
	Mutation	*BRAF* (−)	*BRAF* ( +)
	Annexin A10	(−) ≃ ( +)	(−) < ( +)
	MUC5AC	(−) < ( +)	(−) ≃ ( +)
	DNA methylation	low > intermediate, high	low, high < intermediate
Type B	Histology	SuSA	TSA
	Mutation	*KRAS* ( +)	*KRAS* ( +)
	Annexin A10	(−)	(−) > ( +)
	MUC5AC^†^	(−) > ( +)	(−) < ( +)
	DNA methylation	low, high < intermediate	low, high < intermediate

*MVHP* microvesicular hyperplastic polyp; *TSA* traditional serrated adenoma; *SuSA* superficially serrated adenoma

^*^p < 0.01; ^†^*p* < 0.05

### Differences in the frequencies of annexin A10, mucin marker, and CD10 expression and methylation status in type A2 TSAs between precursor and TSA components

There were no significant differences in the frequencies of annexin A10, mucin marker, and CD10 expression between precursor and TSA components. In addition, no differences in the frequency of methylation status were observed between the two components. The results are summarized in Table 3.

### Differences in the frequencies of annexin A10, mucin marker, and CD10 expression and methylation status in type B TSAs between precursor and TSA components

There was a significant difference in the frequency of MUC5AC expression between the two components (TSA component > precursor component). The results are shown in Table 3.

### Comparison of endoscopic features with pathological findings

Overall, we examined endoscopic findings based on mucus cap, surface color, and pit pattern (available cases, 39 cases). Although there were significant differences in the frequency of mucus positive signs between type A1 and type B TSAs (*p* < 0.05), no differences in the frequency of mucus positive signs were found between type A1 and type A2 TSAs and between type A2 and type B TSAs. Among the three subtypes (type A1, type A2, and type B), no significant differences in the frequency of red color signs between each precursor component or each TSA component were observed. In the pit pattern, type IV-S was a common finding in the TSA component of each subtype. Although all TSAs examined in this study had a type II pit pattern, the frequencies of type II-O patterns were significantly higher in type A1 and type A2 TSAs than in type B TSAs in the precursor component. In addition, there were significant differences in the frequency of the type II-L pit pattern between type A1 and type B TSAs and between type A2 and type B TSAs. The data are depicted in Supplementary Table. Representative images are shown in Supplementary Fig.

## Discussion

TSAs were first described by Longacre and Fenoglio-Preiser in 1990 and have become known as a common type of serrated lesions [4], unlike SSLs, which are considered a new disease. In this study, we examined a series of TSAs with the corresponding precursor component found at base of the polyp and compared the histology, immunohistochemical features, and molecular features of the TSA component with those of the precursor components. The molecular and morphological features of TSA and corresponding precursor components have not been reported in previous studies to date. Our goal was to identify the features of TSA pathogenesis.

Histological findings of the precursor component that suggest progression to the TSA component are critical for evaluating the tumorigenesis of TSAs. Histological differences in the precursor component may reflect different causes of TSA development. Although previous studies have shown that there are two sub-types of TSAs, i.e., BRAF and KRAS subtypes, histological differences in precursor components between the two subtypes have not been fully elucidated. Accordingly, in this study, we showed that there were clear histological differences in precursor components between type A and type B TSAs. Microvesicular HP, which is commonly found in the precursor component of type A TSAs, is expected to progress into either SSLs or TSA [7, 8]. Unfortunately, we could not distinguish whether such histology progressed into TSA or SSLs in this study, based on histological analysis. However, histological features characterizing precursor lesions of type B TSA may be of interest to histopathologists. A recent study showed that superficially serrated adenoma, as described by Hashimoto et al. [23], may have histological findings closely resembling those of the precursor component present in type B TSAs. This finding suggests that type B TSAs may originate from superficially serrated adenoma, which is characterized by mutations in *RNF43* and RSPO fusion/overexpression [23, 24], although these mutations and fusion genes were not examined in the current study. We suggest that there may be two histological pathways, i.e., types A and B, in the tumorigenesis of TSA.

In the current study, we found that the macroscopic type of TSA was closely associated with the subtype of TSA (types A and B) in the TSAs examined in this study. According to this finding, an endoscopist may expect TSA with either *BRAF* mutation (type A) or *KRAS* mutation (type B) based on macroscopic findings. Thus, the macroscopic type of TSA may predict the mutation type of TSA (*BRAF* subtype or *KRAS* subtype). However, it is unclear whether this classification has practical advantages for pathologists or endoscopists. Further studies are needed to demonstrate the clinical usefulness of this classification in routine practice.

In the current study, significant histological differences between type A1 and A2 TSAs were not observed. Although histological differences among these subtypes were not found in the current study, the presence/absence of *BRAF* mutations in the precursor component may play important roles in identifying differences between type A1 and A2 TSAs. Mutations in *BRAF* are known to induce senescence in tumor cells in serrated lesions, suggesting that type A1 TSAs may maintain the stable status of tumor cells owing to *BRAF* mutations and subsequent cellular senescence in TSA cells [25], promoting the progression of TSA. In contrast, type A2 TSAs, which have *BRAF* mutations in the TSA component but not the corresponding precursor component, may play oncogenic roles in the development of type A2 TSAs [26].

Annexin A10 expression is important for differentiation of SSLs from HPs and TSA [27]. Annexin A10, a member of the annexin family, is a calcium- and phospholipid-binding protein that is involved in multiple physiological processes, including growth regulation, cell division, apoptosis, and differentiation [27]. However, the role of annexin A10 expression in TSA is not fully understood. In the current study, we attempted to identify differences in the expression of annexin A10 between the three subgroups. In the current study, although the frequency of annexin A10 expression in the TSA component was significantly higher in type A1 TSAs or type A2 TSAs than in type B TSAs in the two-subgroup statistical analysis, there were significant differences in the frequency of annexin A10 between type A2 and B TSA precursors (type A2 > type B). This finding suggested that expression of annexin A10 played specific roles in the early development of type A2 TSA.

Previous studies have shown that the expression of mucin markers and CD10 contributes to colorectal tumorigenesis [28, 29], although some discrepancies in mucin expression have been reported [30]. MUC2 and MUC5AC are commonly expressed in serrated lesions, including HPs, TSA, and SSLs [9, 31]. However, Owens et al. indicated that MUC6 expression could be used as a specific marker for distinguishing SSLs from HPs [32]. In contrast, Gibson et al. showed that MUC6 is expressed in not only SSLs but also HPs and TSA [31]. Accordingly, MUC6 expression lacks the specificity to distinguish SSLs from other serrated lesions, including HPs and TSA. In the current study, MUC6 expression was not found in type A1, A2, and type B TSAs. Although this finding suggested that MUC6 expression may play a minor role in TSA, the roles of mucins during TSA tumorigenesis remain unclear. Further studies of changes in MUC6 expression during TSA progression from precursor lesions are needed to identify the role of MUC6 in the development of TSA.

In this study, we examined immunohistochemical expression of annexin A10 and methylation status of DNA in each TSA subgroup from corresponding precursor components. Our results showed that annexin A10 expression and moderate DNA methylation were closely associated with the progression of type A1 TSA from the corresponding precursor components. However, annexin A10 expression and DNA methylation status played no fundamental role in the progression of type A2 and B TSAs. According to our current finings, the progression of each TSA subtype may be mediated by different mechanisms related to high expression of annexin A10 and moderate DNA methylation. Additionally, the combination of annexin A10 expression and epigenetic alterations may drive precursor component cells toward the TSA component.

It is important for gastrointestinal endoscopists to compare endoscopic features with the pathological findings of TSAs. First, mucus signs observed on the surface of the TSA component may be a useful marker to distinguish type A1 TSA from type B TSA. Second, no type II-O pits were observed in the precursor component of the type B TSAs examined in the current study. This finding suggested that the absence of type II-O pits may play important roles in the differentiation of type B TSAs from type A TSAs. Finally, the type II-L pattern, occurring in type B TSAs, may characterize the type B TSA examined in the current study. This pit pattern in the precursor component may be closely associated with type B TSA. Detailed observations of the endoscopic features of the precursor component may be helpful for differentiating type B TSAs from type A TSAs.

There were some limitations in the current study. First, the number of patients enrolled in the study was somewhat small. Second, in retrospective cohort studies, a second cohort for validation purposes, in addition to the first cohort, may be needed to confirm the results in patients with TSA. The current study, however, was limited to a single cohort. In the near future, we will attempt to validate the results presented in this study. Second, in this study, cases of cancer in/with TSA were not included. It is important to elucidate the molecular mechanisms mediating the progression to cancer from each TSA subtype. It is well accepted that molecular alterations in cancer originating from TSA include an MSS type accompanied by *TP53* mutations [7, 8]. To resolve this issue, it is necessary to evaluate such cases. However, we were not able to enroll a sufficient number of patients to examine these genetic mechanisms in the current study because cancer in/with TSA is a rare disease in routine pathological diagnosis. Previous studies have shown that the frequency of cancerization occurring in TSA is higher for the KRAS type than for the BRAF type [33]. Thus, additional studies are needed to resolve the molecular mechanisms of cancerization for each type of TSA.

**Fig. 4:**
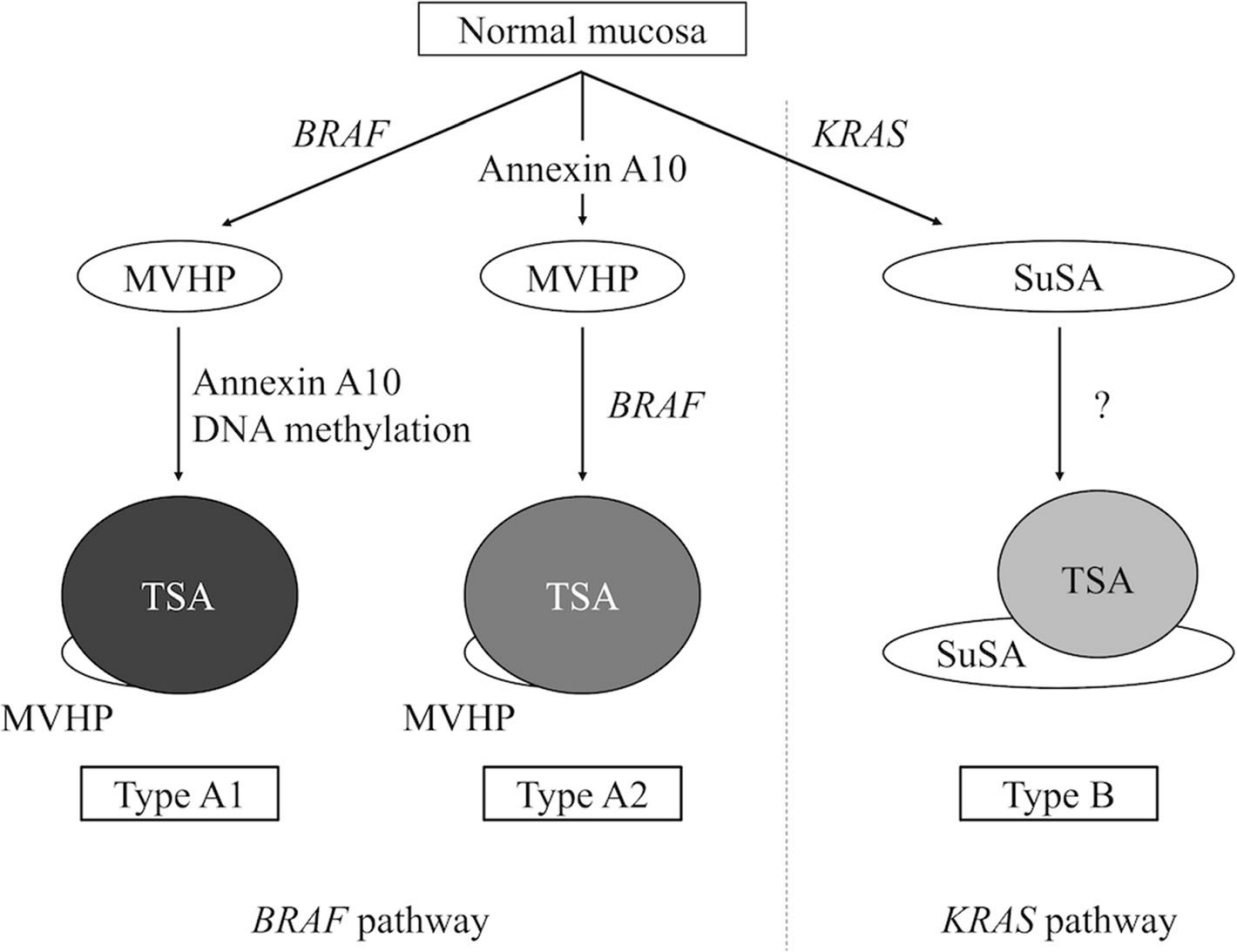
Proposed molecular pathways of neoplastic progression in traditional serrated adenoma

In conclusion, we found that TSAs were represented by two distinct clinicopathological and molecular variants, including type A and B TSAs, which were distinct from *BRAF* and *KRAS* mutations. In addition, we showed that type A TSA could be further subclassified into type A1 and A2 TSAs, characterized by the presence/absence of *BRAF* mutations in the precursor component. Finally, our findings suggested that there were two pathways in which lesions were characterized by potential molecular events and that TSA was a heterogeneous neoplasm with two or three pathways for neoplastic progression. An illustration of the distinct pathological pathways occurring in TSAs, as proposed in the current study, is presented in Fig. 4. Further studies are required to elucidate the molecular mechanisms of potential malignant transformation during TSA progression based on each TSA subtype.

## Electronic supplementary material

Below is the link to the electronic supplementary material.

Supplementary file1 Supplementary Fig. Representative endoscopic images and histological features of type A1, type A2, and type B TSAs. a, Endoscopic images of the TSA component of type A1 TSAs. The type IV-serrated pit pattern is evident (Indigo carmine staining). b. Endoscopic images of the precursor component of type A1 TSAs. The type II pit pattern is clearly demonstrated (Indigo carmine staining). c. The type IV-serrated pits of the lesions were histological features of TSAs. d. The type II pits of the lesion were histological features of microvesicular hyperplastic polyps. e. Endoscopic images of the TSA component of type A2 TSAs. The type IV-serrated pit pattern is evident (Indigo carmine staining). f. Endoscopic images of the precursor component of type A2 TSAs. The type II pit pattern is clearly demonstrated (Indigo carmine staining). g. The type IV-serrated pits of the lesion were a histological feature of TSA. h. The type II pits of the lesion were a histological feature of microvesicular hyperplastic polyps. i, Endoscopic images of the TSA component of type B TSAs. Type IV-serrated pit pattern is evident (Indigo carmine staining). j. Endoscopic images of the precursor component of type B TSAs. The type II-Long pit pattern is clearly demonstrated (Indigo carmine staining). k. The type IV-serrated pits of the lesions were a histological feature of TSA. l. The type II-Long pits of the lesions were a histological feature of superficially serrated adenoma (DOCX 18 kb)

Supplementary file2 (TIF 2767 kb)

## Abbreviations

MSS: Microsatellite stable
MSI: Microsatellite instability
TSA: Traditional serrated adenoma
SSL: Sessile serrated lesion
