# Supplementary material for: Traditional serrated adenoma has two distinct genetic pathways for molecular tumorigenesis with potential neoplastic progression
Source: J Gastroenterol. 2020 Jun 13;55(9):846–57. doi: 10.1007/s00535-020-01697-5 (PMC7452875; doi:10.1007/s00535-020-01697-5)
Supplement: Supplementary file 1 — Supplementary file1 Supplementary Fig. Representative endoscopic images and histological features of type A1, type A2, and type B TSAs. a, Endoscopic images of the TSA component of type A1 TSAs. The type IV-serrated pit pattern is evident (Indigo carmine staining). b. Endoscopic images of the precursor component of type A1 TSAs. The type II pit pattern is clearly demonstrated (Indigo carmine staining). c. The type IV-serrated pits of the lesions were histological features of TSAs. d. The type II pits of the lesion were histological features of microvesicular hyperplastic polyps. e. Endoscopic images of the TSA component of type A2 TSAs. The type IV-serrated pit pattern is evident (Indigo carmine staining). f. Endoscopic images of the precursor component of type A2 TSAs. The type II pit pattern is clearly demonstrated (Indigo carmine staining). g. The type IV-serrated pits of the lesion were a histological feature of TSA. h. The type II pits of the lesion were a histological feature of microvesicular hyperplastic polyps. i, Endoscopic images of the TSA component of type B TSAs. Type IV-serrated pit pattern is evident (Indigo carmine staining). j. Endoscopic images of the precursor component of type B TSAs. The type II-Long pit pattern is clearly demonstrated (Indigo carmine staining). k. The type IV-serrated pits of the lesions were a histological feature of TSA. l. The type II-Long pits of the lesions were a histological feature of superficially serrated adenoma (DOCX 18 kb) [file 535_2020_1697_MOESM1_ESM.docx]

|  |  | Type A1 (%) | Type A2 (%) | Type B (%) | *P* value |
| --- | --- | --- | --- | --- | --- |
| Total |  | 18 (100) | 6 (100) | 15 (100) |  |
| Mucus | Negative | 2 (11.1) | 2 (33.3) | 9 (60.0) | 0.0077 |
|  | Positive | 16 (88.9) ^†^ | 4 (66.7) | 6 (40.0) ^†^ |  |
| Color |  |  |  |  |  |
| TSA component | NC | 0 (0) | 0 (0) | 0 (0) | 1.0000 |
|  | RC | 18 (100) | 6 (100) | 15 (100) |  |
| Precursor component | NC | 15 (83.3) | 6 (100) | 15 (100) | 0.2644 |
|  | RC | 3 (16.7) | 0 (0) | 0 (0) |  |
| Pit pattern |  |  |  |  |  |
| TSA component | Type IV-S | 18 (100) | 6 (100) | 15 (100) | 1.0000 |
| Precursor component | Type II | 11 (61.1) | 3 (50.0) | 6 (40.0) | 0.0022 |
|  | Type II-O | 5 (27.8) ^†^ | 3 (50.0) ^†^ | 0 (0) ^†, †^ |  |
|  | Type II-L | 2 (11.1) * | 0 (0) ^†^ | 9 (60.0) *^, †^ |  |

Supplementary Table. Comparison of endoscopic findings among type A1, type A2, and type B TSAs

*, *p* < 0.01; ^†^, *p* < 0.05

TSA, traditional serrated adenoma; NC, normal-colored or pale in color of mucosa; RC, Reddish change in color; Type II-O, type II-Open; Type II-L, type II-Long; Type IV-S, type IV-Serrated
