# Supplementary figures and images for: Traditional serrated adenoma has two distinct genetic pathways for molecular tumorigenesis with potential neoplastic progression
Source: J Gastroenterol. 2020 Jun 13;55(9):846–57. doi: 10.1007/s00535-020-01697-5 (PMC7452875; doi:10.1007/s00535-020-01697-5)

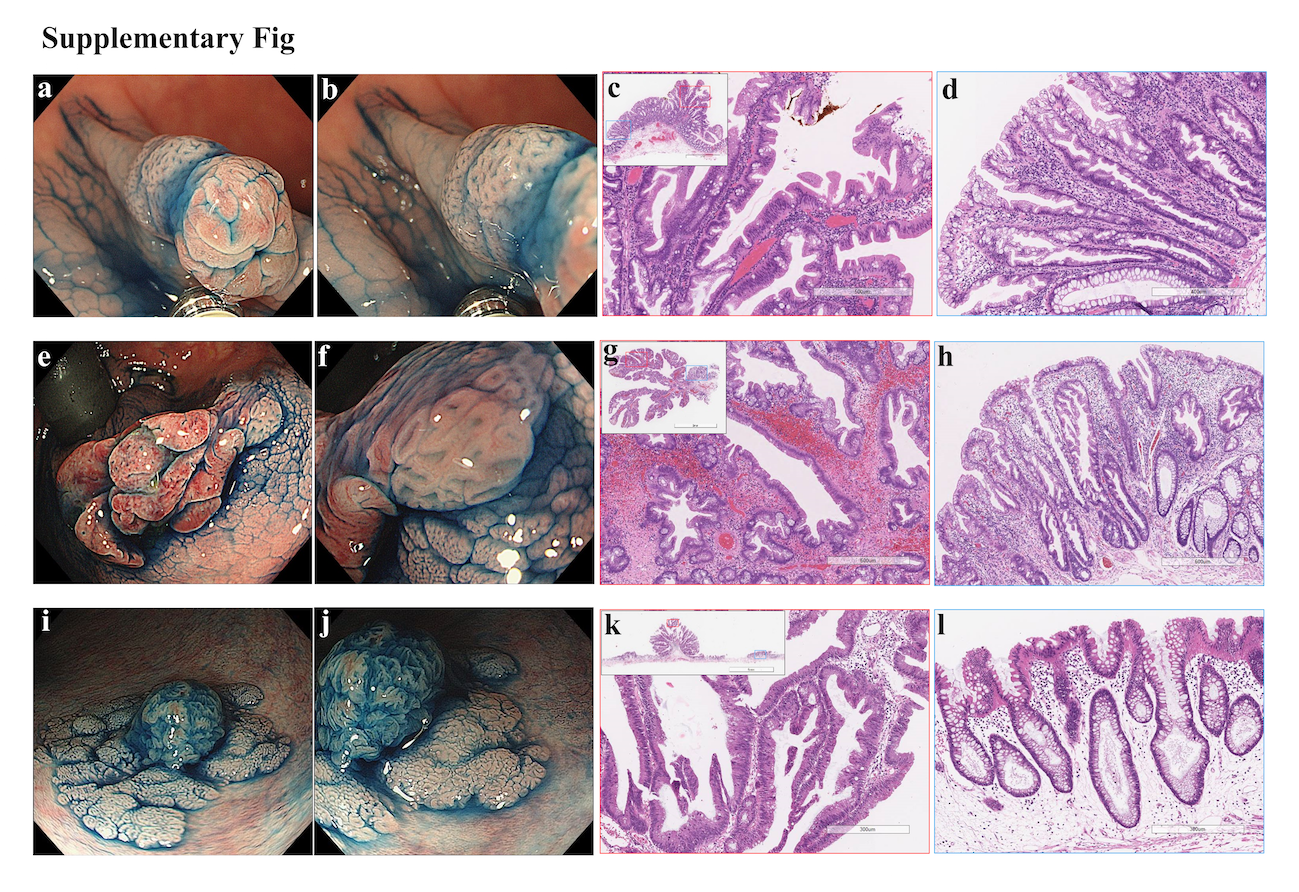

Supplement: Supplementary file 2 — Supplementary file2 (TIF 2767 kb) [file 535_2020_1697_MOESM2_ESM.tif]
